# Effect of interval training intensity on fat oxidation, blood lactate and the rate of perceived exertion in obese men

**DOI:** 10.1186/2193-1801-2-532

**Published:** 2013-10-17

**Authors:** Shaea A Alkahtani, Neil A King, Andrew P Hills, Nuala M Byrne

**Affiliations:** School of Exercise and Nutrition Sciences, Queensland University of Technology, Australia & University of Dammam, P.O. Box 2375, Dammam, 31451 Saudi Arabia; School of Exercise and Nutrition Sciences & Institute of Health and Biomedical Innovation, Queensland University of Technology, Brisbane, Australia; Mater Medical Research Institute and Griffith Health Institute, Griffith University, Brisbane, Australia

**Keywords:** Interval exercise, Fat_max_, Maximal fat oxidation

## Abstract

**Purpose:**

The objectives of this study were to examine the effect of 4-week moderate- and high-intensity interval training (MIIT and HIIT) on fat oxidation and the responses of blood lactate (BLa) and rating of perceived exertion (RPE).

**Methods:**

Ten overweight/obese men (age = 29 ±3.7 years, BMI = 30.7 ±3.4 kg/m^2^) participated in a cross-over study of 4-week MIIT and HIIT training. The MIIT training sessions consisted of 5-min cycling stages at mechanical workloads 20% above and 20% below 45%VO_2peak_. The HIIT sessions consisted of intervals of 30-s work at 90%VO_2peak_ and 30-s rest. Pre- and post-training assessments included VO_2max_ using a graded exercise test (GXT) and fat oxidation using a 45-min constant-load test at 45%VO_2max_. BLa and RPE were also measured during the constant-load exercise test.

**Results:**

There were no significant changes in body composition with either intervention. There were significant increases in fat oxidation after MIIT and HIIT (p ≤ 0.01), with no effect of intensity. BLa during the constant-load exercise test significantly decreased after MIIT and HIIT (p ≤ 0.01), and the difference between MIIT and HIIT was not significant (p = 0.09). RPE significantly decreased after HIIT greater than MIIT (p ≤ 0.05).

**Conclusion:**

Interval training can increase fat oxidation with no effect of exercise intensity, but BLa and RPE decreased after HIIT to greater extent than MIIT.

## Introduction

Obesity is closely related with development of insulin resistance, and the relationship is, in part, mediated through impairment of fat oxidation. Exercise is advocated as a strategy to improve skeletal muscle fat oxidation (Koves et al. 2008; Rogge 2009). It has been demonstrated that the uptake and oxidation of free fatty acid increases during moderate-intensity exercise, whereas the delivery, uptake and oxidation of free fatty acid is limited during high-intensity exercise (Frayn 2010). In contrast, high-intensity exercise results in greater post-exercise fat oxidation than is found with moderate-intensity exercise (Pillard et al. 2010). While the differential impact of moderate versus high-intensity continuous training on fat oxidation has been well established from several medium-term studies (Amati et al. 2008; Maffeis et al. 2005; Van Aggel-Leijssen et al. 2002; Zarins et al. 2009), it is not clear if the same responses are demonstrated during interval training.

Previous studies have not compared the effect of different exercise intensities of interval training on fat oxidation. Comparison has been made between 4-week moderate-intensity continuous exercise at intensity that elicits maximal fat oxidation (FAT_max_) versus moderate-intensity interval training at ±20%FAT_max_ in obese adults. The findings revealed that exercise-induced fat oxidation increased after continuous training whereas it did not change after interval training (Venables and Jeukendrup 2008). Another study has compared six sessions of high volume, moderate-intensity continuous exercise at 65%VO_2max_ (total time commitment was 10.5 h) with low volume, supramaximal interval training (repeated Wingate Test of total time commitment of 2.5 h), and found similar adaptations in muscle oxidative capacity after both trainings (Gibala et al. 2006). The comparisons between post and pre high-intensity interval training were also tested in healthy adults, and findings included a significant increase in exercise-induced fat oxidation after seven sessions of interval training at 90%VO_2max_ (Talanian et al. 2007) and significant increases in the maximal activity of cytochrome c oxidase (COX) and the protein content of COX subunits II and IV after six sessions of interval training at VO_2max_ (Little et al. 2010). Another study compared pre- with post-assessment of 6-session supramaximal interval exercise intervention in sedentary overweight/obese men (Whyte et al. 2010). In this study, there was a significant increase in resting fat oxidation at 24-h post training, but the significance did not remain at 72-h post training. The current study design uses different intensities of interval training with the same total mechanical work.

Evidence suggests that time spent during interval training at maximal velocity or maximal power output is a key factor in improving VO_2max_ (Billat 2001; Helgerud et al. 2007). However, obese individuals may find training at maximal levels are less tolerable compared with moderate-intensity training. For example, imposing a speed at just 10% higher than the self-selected speed reduced the pleasure of exercise in obese compared to non-obese sedentary women (Ekkekakis and Lind 2006). Monitoring BLa and RPE during training sessions will demonstrate the level of physical stress that participants experience during training (Norton et al. 2010). Recently, a comparison was made between three training programs, interval training of 4 × 8 min at 90%VO_2max_, interval training of 4 × 4 min at VO_2max_ and interval training of 4 × 16 min at LT in moderately trained cyclists. Interval training that included 4 × 8 min bouts increased the workload at a constant-load exercise test and reduced RPE during 7-week training sessions greater than the other training groups (Seiler et al. 2011). Unlike trained individuals, obese participants may not be able to tolerate interval training at high-intensity levels for 4–8 min. Therefore, if high-intensity short-stages interval training (i.e., training at 90%VO_2max_ for 30-s intervals) can increase aerobic capacity and decrease BLa and RPE, it will provide obese individuals with an easier training option than high-intensity long-stages interval training.

The primary objective of this study was to compare four weeks of moderate-intensity with high-intensity interval training (MIIT and HIIT) on exercise-induced fat oxidation. The second aim was to compare the effect of interval training intensity on BLa and RPE.

## Methods

### Participant characteristics

Participants included 10 sedentary overweight/obese men. The characteristics of participants were: age (29 ±3.7 years), weight (88.6 ±7.6 kg), BMI (30.7 ±3.4 kg/m^2^), fat mass (31.2 ± 4.7%body weight) and VO_2peak_ (28.7 ±3.4 ml/kg/min). Participants were recruited from the staff and student population at the Queensland University of Technology (QUT) and the Brisbane metropolitan region via e-mail and flyers posted on community noticeboards. A written informed consent was signed and the participant was asked to gain medical clearance from a general practitioner prior to undertaking the study. The study protocol was approved by the Human Research Ethics Committee at QUT (HREC No. 1000000160).

### Experimental design and assessment tests

The study employed a cross-over design, and involved two 4-week exercise training interventions consisted of 12 cycling sessions in each intervention (moderate-intensity interval training (MIIT) or high-intensity interval training (HIIT)), separated by 6-week detraining wash-out.

Three assessments were taken at week 0 (pre-intervention) and week 4 (post-intervention). The first assessment was blood collection, and on the following day, participants performed MFO and VO_2max_ test on a braked cycle ergometer (Monark Bike E234, Monark Exercise AB, Sweden). The MFO and VO_2max_ test was performed after overnight fasting. The constant-load exercise testing was performed in the afternoon at approximately 1 pm, 48 h after the MFO and VO_2max_ test, and participants were asked to have their breakfast at least 5 h before the test.

Participants were asked to maintain their habitual diet, activity and alcohol intake during their participation in the current project. On the pre-assessment day, participants were asked to abstain from any kind of exercise or physical work, and to abstain from consuming alcohol, caffeine or salty food. The participants recorded their food and drink intake on the day before the first assessment, and were asked to have the same food before all remaining assessment tests.

#### MFO and VO_2max_ test

The FAT_max_ graded cycle ergometry protocol was discontinuous, with participants cycling at 35 W for 4 min followed by a 4-min rest interval. At the end of the rest interval the work rate was increased by 17.5 W and the participant cycled at the new workload for 4 min. The discontinuous sequence of 4-min work-rest stages with 17.5 W increments in workload continued until the workload at which RER reached 1.0 and remained above 1.0 during the final 2 min of exercise. After a 4-min rest, participants commenced the second phase of the test designed to determine maximal aerobic power. Participants cycled for a minute at a workload two increments lower than the intensity at which an RER of 1.0 was reached, after which the mechanical work was increased by 17.5 W every minute until volitional exhaustion. This protocol has been adapted from Achten et al. (2002), which has been used in the obese men population (Roffey et al. 2007).

#### Constant-load exercise test

The constant-load exercise test was performed at 45%VO_2max_ for 45 min at cadence of 70 rpm, followed by 1 h recovery. Expired gas was collected and heart rate was monitored during 5 min resting phase (−5 to 0), intermittently between minutes (10 to 15), (25 to 30) and (40 to 45) during exercise phase and intermittently between minutes (+15 to +30) and (+45 to +60) during the recovery phase. In the recovery phase, participants were seated calmly on a chair, and were not allowed to do any activities. Fingertip blood lactate samples were collected, and Borg scale 6–20 was undertaken at minute 0, 15, 30, 45, +30 and +60.

### Training sessions

All participants participated in 12 supervised cycling exercise sessions, three exercise sessions per week. The duration of exercise started with 30 min in the first week, and increased by 5 min every week to reach 45 min in the last week. All exercise sessions started with unloaded 5-min warm-up cycling and ended with unloaded 5-min cool-down cycling at a cadence of 70 rpm. The actual mechanical work including total distance (km) and HR were monitored in all 12 exercise sessions. In the first exercise sessions of each week, HR, RPE and fingertip BLa samples were collected and recorded before the exercise, at minutes 5, 15 and 30 and at the end of the exercise session.

Moderate-intensity interval exercise (MIIT) was determined at 45%VO_2max_, and high-intensity interval exercise (HIIT) was determined at 90%VO_2max_. The simple linear regression between VO_2_ and workload was performed and the slope and intercept were calculated to determine the individual workloads that represent 45 and 90%VO_2max_. Participants randomly proceeded to either MIIT or HIIT in a counterbalanced order.

The MIIT session consisted of 5-min cycling stages at ±20% of mechanical work at 45%VO_2max_. After the workload was determined at 45%VO_2max_, the workload was multiplied by ± 0.2 to determine the ±20% workload of 45%VO_2max_. The participants started every exercise session with a workload at 20% above 45%VO_2max_ for 5 min, followed by 5 min with a workload at 20% below 45%VO_2max_, and consequently changed until the end of exercise session.

The HIIT session consisted of a 30-s work at 90%VO_2_max and 30-s passive recovery interval rests per minute. The 30-s exercise was repeated 30 times per 30-min session in the first week and was repeated 45 times per 45-min session in the fourth week. The researcher supervised all sessions and encouraged participants to stop at 30 s and start at the beginning of the subsequent minute till the end of the exercise. Participants were instructed to monitor time and cadence on the fitness screen in the handlebars, and to cycle as fast as possible at the beginning of each minute so as to get to the goal cadence of 70 rpm within 2 s, and then were required to keep their cadence at 70 rpm for the entire exercise time.

### Data management of indirect calorimetry

The Parvo Medics Analyser Module (TrueOne® 2400, Metabolic Measurement System, Parvo Medics, Inc. USA) was used in the measurement of respiratory gas exchange, and the calibration of the system was undertaken prior to each test. Expired breaths were collected, and VO_2_, VCO_2_, RER and HR were averaged for every 30 s automatically via the Parvo Medics Analyser. Data were then exported to an Excel file.

During the MFO and VO_2max_ test, the last 2-min of each exercise stage where RER <1.0 during the discontinuous phase were averaged. The last 30 s of each minute during the continuous stage were used to attain VO_2_, VCO_2_, RER and HR. Workload, VO_2_, VCO_2_, HR, RER, BLa were calculated at the point of VO_2max_. Five threshold criteria were used to determine maximal aerobic performance. Individual 50% increment of VO_2_ was used to calculate the VO_2_ plateau, and its average was 1.25 ±0.3 ml/kg/min (R^2^ = 0.96 ±0.09) in pre-MIIT and HIIT.

During the constant-load test, the average of 5 min during rest, 5 min at minute (10 to 15) and (25 to 30) and (40 to 45) during exercise and 15 min at minute (+15 to +30) and (+45 to +60) during the recovery period were calculated. Rate of fat oxidation was calculated using stoichiometric equations of the thermal equivalents of oxygen for non-protein RQ (Frayn 1983).

### Statistical analysis

Data were presented as mean values and standard error of mean (SEM), unless otherwise indicated. A mixed linear model univariate ANOVA was used to assess the interaction between intensity (MIIT and HIIT) and time (weeks 0 and 4) on fat oxidation and physiological variables.

In the constant-load exercise test, time point (data collected during the test at 15, 30 and 45 min) was added as a fixed factor to univariate analysis, but the interaction of this factor with intensity or time was not computed. The training order in the cross-over design was added as a fixed factor, and the interaction between this factor and intensity and time was computed. In the training sessions, period (data collected during weeks 1,2,3 and 4) was added as a fixed factor to univariate analysis.

The Microsoft Excel program (version 12.0, 2007, Microsoft Corporation, Seattle, WA, USA) was used to compile the main results. Statistical analyses were carried out with SPSS for Windows (version 18.0.1, 2010, PASW Statistics SPSS, Chicago, IL, USA).

## Results

### Description of exercise training sessions

The workload and time of exercise sessions were matched between MIIT and HIIT. Total duration of 12 training session was 450 min, and average workload was 76 ±4. As a result, average distance and time of cycling interval stages per session during HIIT was significantly lower than MIIT (P ≤ 0.001). Total energy expenditure for 12 training sessions was predicted using participants’ energy expenditure from the constant-load exercise test at week 0, and was approximately 6.4 kcal/min × 450 min = 2880 kcal.

### Body composition and VO_2peak_

There were no significant effects of intensity and time on body composition (Table 1). There were significant effects of time on VO_2peak_ (P ≤ 0.05) and W_max_ (P ≤ 0.001), whereas there were no significant effects of time on RER_peak_, HR_peak_, BLa_peak_ and RPE_peak_. With reference to the GXT, five threshold criteria were used to determine maximal aerobic performance. Three out of 10 participants in MIIT and five out of 10 participants in HIIT reached a plateau in VO_2max_; nine reached their predicted HR_max_ prior to both interventions. All participants reached the RER_peak_, BLa_peak_ and RPE_peak_ criteria prior to MIIT and HIIT. The post-measures were comparable with pre-measures except for two participants who did not reach RPE_peak_ after HIIT although they reached other criteria.

**Table 1:** **Body composition and VO**_**2peak**_ **at weeks 0 and 4 in MIIT and HIIT**

Variables	MIIT			HIIT		
	Week 0	Week 4	Change (%)	Week 0	Week 4	Change (%)
Weight (kg)	88.6 ± 2.4	88.9 ± 2.4	+0.3 (0.3)	89.2 ± 2.5	89.3 ± 2.4	+0.1 (0.1)
Fat mass (%)	34.9 ± 1.1	33.7 ± 1.5	−1.2 (−3.4)	34.5 ± 1.2	34.8 ± 1.3	+0.3 (0.8)
VO_2peak_ (ml/kg/min)	28.7 ± 1.1	29.5^*^ ± 1.1	+0.8 (2.8)	27.0 ± 1.1	28.9^*^ ± 0.9	+1.9 (7.0)
W_max_ (W)	204 ± 6	215^**^ ± 8	+11.4 (5.6)	20.0 ± 7	222^**^ ± 10	+21.6 (10.8)

Footnote:

MIIT: moderate-intensity interval training; HIIT: high-intensity interval training; VO_2peak_: peak oxygen consumption; W_max_: maximal workload.

*Significant difference between weeks 4 and 0 (P ≤ 0.05).

**Significant difference between weeks 4 and 0 (P ≤ 0.01).

Data expressed as mean ± SEM, absolute and percentage differences.

### Fat oxidation

**Figure 1:**
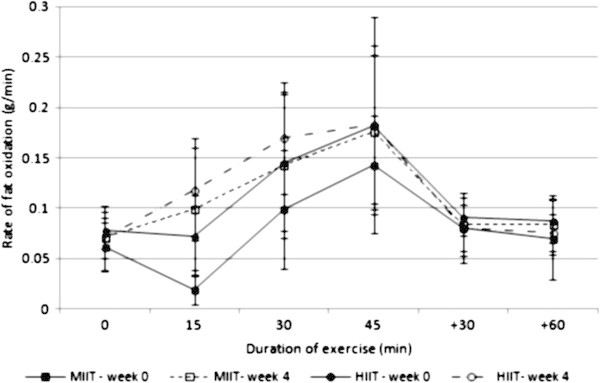
**Rate of fat oxidation during the constant-load exercise test at week 0 (solid line) and week 4 (dotted line) in MIIT (square) and HIIT (circle); data represented as mean ± SD.** The effect of time on fat oxidation was significant (p ≤ 0.001). MIIT: moderate-intensity interval training; HIIT: high-intensity interval training.

The effect of time on the rate of exercise-induced fat oxidation was significant (P ≤ 0.001), whereas the interaction between intensity and time on the rate of fat oxidation was not significant. As the effect of intensity on fat oxidation was also significant (P ≤ 0.01), the rates of fat oxidation during 15, 30 and 45 min were averaged and the training order in the cross-over design was added as a fixed factor. There was no significant effect of training order on fat oxidation, and the interaction between training order and time and the interaction between training order and intensity were not significant. In addition, paired t test revealed that there was no significant difference between MIIT and HIIT in delta fat oxidation (i.e., delta represents difference between average fat oxidation in weeks 4 and 0). Figure 1 shows the responses of fat oxidation during the constant-load exercise test in MIIT and HIIT.

The effect of time on MFO was significant (P ≤ 0.01), but the interaction between intensity and time was not significant. MFOs for pre-intervention were 0.13 ±0.07 g/min and 0.10 ±0.10 g/min, and post-intervention were 0.17 ±0.09 g/min and 0.18 ±0.06 g/min for MIIT and HIIT respectively. Post hoc power analysis, computed using partial eta squared = 0.038 and correlation among repeated measures = 0.6, revealed that the effect size of the interaction between intensity and time on fat oxidation was 0.19 and power was 0.30. Thirty participants are required to attain power of 0.80.

### The responses of BLa and RPE

**Figure 2:**
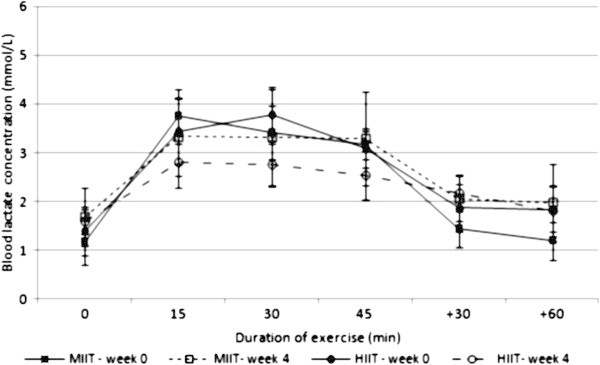
**Concentration of blood lactate during the constant-load exercise test at week 0 (solid line) and week 4 (dotted line) in MIIT (square) and HIIT (circle); data represented as mean ± SEM.** There was a significant effect of time (p ≤ 0.05). The interaction between intensity and time was not significant (P = 0.09). MIIT: moderate-intensity interval training; HIIT: high-intensity interval training.

**Figure 3:**
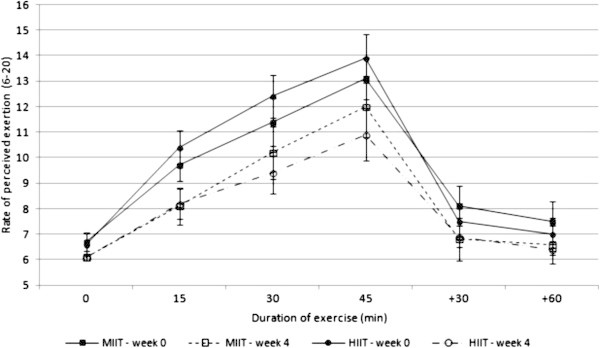
**Rate of perceived exertion (RPE) during the constant-load exercise test at week 0 (solid line) and week 4 (dotted line) in MIIT (square) and HIIT (circle); data represented as mean ± SEM.** There was a significant effect of time (p ≤ 0.001). The interaction between time and intensity was significant (p ≤ 0.05). MIIT: moderate-intensity interval training; HIIT: high-intensity interval training.

BLa and RPE significantly decreased during the constant-load exercise test after MIIT and HIIT, revealing a significant effect of time (P ≤ 0.05 and 0.001 for BLa and RPE respectively). BLa was lower during HIIT than MIIT but the interaction between intensity and time was not significant (P = 0.09). The interaction between time and intensity on RPE was significant (P ≤ 0.05) revealing a greater decrease after HIIT than MIIT (−1.3 unit in MIIT vs −2.7 unit in HIIT). Figures 2 and 3 show the responses of BLa and RPE during the constant-load exercise test at weeks 0 and 4 in MIIT and HIIT.

**Figure 4:**
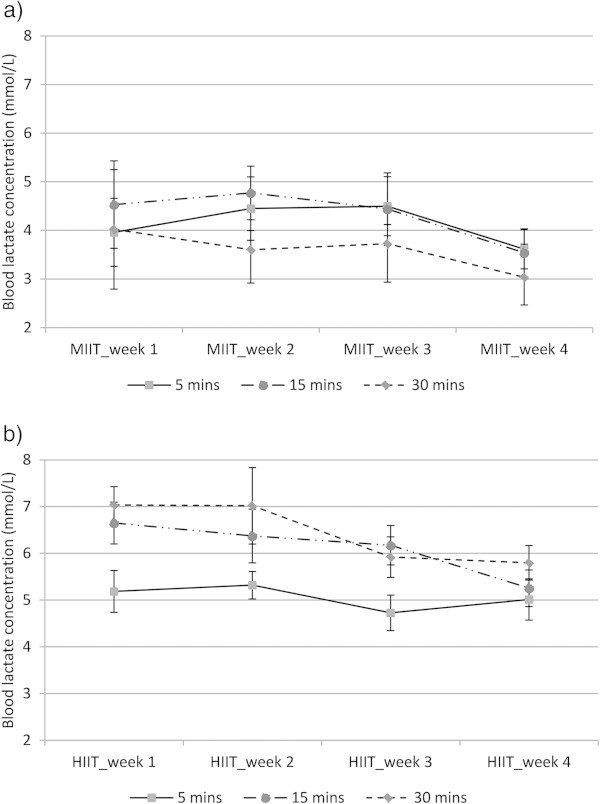
**The concentration of blood lactate (BLa) during 4-week training of MIIT (a) and HIIT (b); data collected in the first exercise session of every week; data represented as mean ± SEM.** Note: workload at 30 mins of MIIT was lower than workload at 5 and 15 mins of MIIT. There was a significant effect of training period (weeks) (p ≤ 0.001). MIIT-1-4: moderate-intensity interval training at weeks 1, 2, 3 and 4; HIIT-1-4: high-intensity interval training at week 1, 2, 3 and 4.

**Figure 5:**
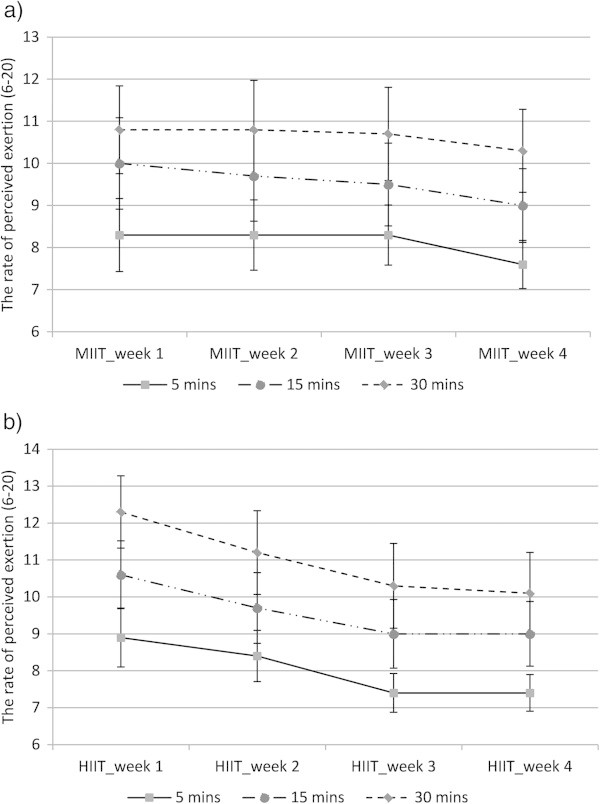
**The rate of perceived exertion (RPE) during 4-week training of MIIT (a) and HIIT (b); data collected in the first exercise session of every week; data represented as mean ± SEM.** Note: workload at 30 mins of MIIT was lower than workload at 5 and 15 mins of MIIT. There was a significant effect of training period (weeks) (p ≤ 0.001), and an interaction between intensity and training period (weeks) on RPE (p ≤ 0.05). MIIT-1-4: moderate-intensity interval training at weeks 1, 2, 3 and 4; HIIT-1-4: high-intensity interval training at week 1, 2, 3 and 4.

BLa, HR and RPE were monitored and collected at the first session of every week in minutes 5, 15 and 30 during MIIT and HIIT. These variables were also collected at the end of exercise bouts, but as the workload at the end of MIIT was 20% either above or below 45%VO_2peak_, data at this point was not included in statistical analysis. The values of BLa and HR were significantly higher during HIIT than MIIT (P ≤ 0.001), but there was no significant difference in RPE between MIIT and HIIT. While BLa and HR corresponded to the workload during MIIT, RPE significantly increased with exercise duration independent of intensity (P ≤ 0.001). BLa, HR and RPE declined during the 12-exercise sessions in MIIT and HIIT revealing significant effects of training period (weeks) (P ≤ 0.001). Post hoc comparisons revealed that significant decreases in BLa and HR were detected in week 4 (after nine sessions) compared with weeks 1 and/or 2 (P ≤ 0.001), whereas the statistical decrease in RPE was detected from week 3 (after six sessions) compared with week 1 (P ≤ 0.001). Figures 4 and 5 show the responses of BLa and RPE respectively during 4-week training sessions of MIIT (a) and HIIT (b).

## Discussion

### Fat oxidation

The main finding of this study was that 12 sessions of interval training undertaken within four weeks significantly increased medium-term exercise-induced fat oxidation in overweight/obese men, with no difference identified between training intensities. In concordance with HIIT, Talanian et al. (2007) found that seven sessions of long work-to-rest ratio of high-intensity interval training at 90%VO_2max_ significantly increased exercise-induced fat oxidation in moderately active women. To the contrary with MIIT, Venables and Jeukendrup (2008) did not find an increase in exercise-induced fat oxidation after 12 sessions of alternated 5-min exercise at ±20% of FAT_max_ in obese men, whereas fat oxidation increased after continuous moderate-intensity training.

While there is a lack of studies that compare different intensities of interval training, studies that used continuous training did not find moderate-intensity training to be more favourable for obese individuals to increase fat oxidation. For example, Van Aggel-Leijssen et al. (2002) found a significant increase in exercise-induced fat oxidation after moderate-intensity aerobic training at 40%VO_2max_ for 12 weeks in obese men, whereas no change was found after high-intensity aerobic training at 70%VO_2max_. In this study, there was no significant difference between groups in the change of fat oxidation. The participants also started the moderate-intensity training with a lower level of fat oxidation than high-intensity training, whereas the levels of post-training fat oxidation were comparable between moderate and high-intensity training groups. Therefore, there are no consistent findings supporting the view that moderate-intensity training can increase fat oxidation to a greater extent than high-intensity exercise in the obese population.

The similar effectiveness of moderate- and high-intensity interval training on fat oxidation is supported by theoretical explanation of the mechanism of mitochondrial signalling pathways that target PGC-1α mRNA transcription (Laursen 2010). The high-intensity of endurance training such as HIIT can increase the mitochondrial oxidative capacity and consequently improve fat oxidation potential through the AMP-activated protein kinase pathway, and the high-volume of endurance training such as MIIT can increase the mitochondrial oxidative capacity through the calcium-calmodulin kinases pathway (Laursen 2010). Wang et al. (2009) compared high-intensity interval training at 120%VO_2max_ for 12 s with 18-s recovery intervals at 20%VO_2max_ and moderate-intensity continuous exercise at 67%VO_2max_ for 90 min in sedentary individuals, and found identical increases in the mRNA content for major regulators of mitochondrial biogenesis (peroxisome proliferator- activated receptor and PGC-1α) and lipid metabolism (pyruvate dehydrogenase kinase isozyme 4).

It is important to consider that the change in substrate oxidation was small. The participants predominantly relied on CHO (70–75%EE) during the acute constant-load exercise test at weeks 0 and 4 of MIIT and HIIT. This small change in substrate oxidation after the intervention was similar to a previous study in the obese. For example, fat oxidation was 0.11 g/min and CHO oxidation was 0.08 g/min at the baseline, and after six sprint interval sessions fat oxidation was 0.13 and 0.12 g/min and CHO oxidation was 0.03 and 0.05 g/min at 24-hr and 72-hr after the intervention in obese men (Healy et al. 2011). It was reported that sedentary obese individuals have an impaired ability to oxidise fat in skeletal muscles (Corpeleijn et al. 2009). It is important that fat reserves are very large in the human body (Flatt, 1995; Galgani & Ravussin 2008). Therefore, it is not expected that the increase in fat oxidation after HIIT and MIIT per se will help in burning body fat mass. The interaction between the increase in fat oxidation and conservation of muscle glycogen and between fat oxidation and eating behaviour could be the important goal in increasing fat oxidation for weight management. The increase in fat oxidation after MIIT and HIIT is a marker which reflects the stability in body weight in the long-term. It was reported that the low rate of fat oxidation can lead to the increase in body weight due to the susceptibility to store excess energy as fat (fat balance) (Zurlo et al. 1990) and to have a greater tendency to deplete glycogen (CHO balance) (Flatt 1987; Pannacciulli et al. 2007).

### Responses of BLa and RPE

The curve of BLa during the constant-load test in pre and post MIIT and HIIT demonstrated three main trends. First, it decreased in the pre- and post-intervention after the initial increase during the session, representing a normal stabilisation during moderate-intensity exercise, and demonstrating that pyruvate is consumed aerobically more than its generation via anaerobic glycolysis (Beneke et al. 2011). Second, it increased in the initial 15-min exercise then started to decrease in the pre-intervention because of the initial oxygen deficit, but it did not increase at the beginning of the constant-load exercise test in the post-HIIT, which could be attributed to the rapid increase in VO_2_ uptake in the onset of exercise and reflects an improvement in aerobic capacity after HIIT (Jones and Carter 2000). Lastly, the curves of BLa were different between MIIT and HIIT in the pre-intervention, but they were apparently similar in the post-intervention, and BLa at HIIT was lower than MIIT. The similarity between MIIT and HIIT in the shape of BLa curve supports the proposal of the importance of aerobic training to improve BLa, and the lower concentration of BLa after HIIT than MIIT supports the proposal of the importance of intensity in improving BLa (Beneke et al. 2011).

The reduction in BLa was associated with the decrease in RPE. The relationship between BLa and RPE at high-intensity levels has been found in some previous studies (Seiler et al. 2011). RPE is mediated by psychological factors during moderate-intensity, and mediated by physiological variables during high-intensity (Coutts et al. 2009; Pires et al. 2011). Therefore, the association between BLa and RPE after high-intensity training reflects peripheral metabolic adaptations that collectively attenuate RPE (Tucker 2009; Hampson et al. 2001). These peripheral adaptations include reduced glycogenolysis and reduced lactate accumulation, which were detected after six weeks of interval training at 90%VO_2peak_ in untrained participants (Perry et al. 2008).

Few studies have investigated the time course of physiological adaptation during aerobic training. For example, HR during exercise in young and older women significantly decreased by 10 and 4 beats/min respectively at week 3 (after nine sessions) of training at 70%VO_2max_, with no further changes in weeks 6, 9 and 12 (Murias et al. 2010). A significant reduction in submaximal HR was found after three sessions performed in one week, and a further decrease was found after three weeks, but BLa did not change during an incremental test at weeks 1 and 3 after training at 70%VO_2max_ for 45 min per session, and LT did not change after one week of training but significantly increased at week 3 in sedentary young men (Ziemba et al. 2003). The key parameters of aerobic fitness and endurance performance are maximal and submaximal VO_2_ and BLa (Jones and Carter 2000). Therefore, it is important to compare the time course that is required to reduce BLa with the time course that is required to improve VO_2_ in previous studies. Several studies have found that aerobic high-intensity interval training of six and seven sessions improved VO_2_max (Little et al. 2010; Talanian et al. 2007). Nine sessions of continuous aerobic training at 70%VO_2_max improved VO_2_max in older and young men (Murias et al. 2010). VO_2_max did not change after one week of aerobic training at 70%VO_2_max for 45 mins three times per week, but significantly increased after three weeks, and submaximal O_2_ uptake did not change at weeks 1 and 3 (Ziemba et al. 2003). Collectively, the current result and previous studies suggest that nine sessions are adequate to improve physiological markers related to aerobic performance.

In conclusion, 12 sessions of MIIT and HIIT exercise performed in four weeks resulted in statistically significant improvements in exercise-induced fat oxidation, with improvements being independent of exercise intensity. BLa and RPE decreased during the constant-load test and training sessions of HIIT to a greater extent than MIIT. This finding reinforces previous findings which suggested the difficulty of anaerobic interval training (Billaut, et al. 2003), and found a decrease in work efficiency after this model (McCartney, et al. 1986). The repeated Wingate-protocol model was used among the obese, and the researchers agreed that by manipulating supramaximal exercise in the obese population may increase the risk of side effects (Whyte et al. 2010). On the other hand, aerobic interval training was more tolerable than submaximal continuous training. For example, aerobic interval exercise as 15 secs at 100%VO_2_max with 15-sec interval rests (Guiraud et al. 2010) or alternate by ±7%HRmax around 67%HRmax (Kang et al. 2005) were perceived as easier than matched continuous workload as indicated by RPE. Therefore, aerobic interval exercise that is performed at intensities below VO_2_max could be the appropriate training option among obese individuals, and can be recommended for healthy obese men. Future studies may investigate the effect of different lengths of high-intensity interval training on fat oxidation, BLa and RPE.

## Authors’ information

Nuala M. Byrne, Neil A. King, Andrew P. Hills are co-authors.
